# Moisture-driven, self-powered noncontact sensing interfaces via turbulence-tailored hygroelectronic effect

**DOI:** 10.1126/sciadv.aee7050

**Published:** 2026-04-17

**Authors:** Daozhi Shen, Haotian Luo, Gangli Zhao, Zechao Han, Zhengsheng Yang, Xinyi Le, Yanjie Su, Runze Ma, Limin Zhu

**Affiliations:** ^1^ School of Mechanical Engineering, Shanghai Jiao Tong University, Shanghai 200240, China.; ^2^ National Center for Translational Medicine, Shanghai Jiao Tong University, Shanghai 200240, China.; ^3^ School of Logistics Engineering, Shanghai Maritime University, Shanghai 201306, China.; ^4^ Department of Automation, Shanghai Jiao Tong University, Shanghai 200240, China.; ^5^ National Key Laboratory of Advanced Micro and Nano Manufacture Technology, Shanghai Jiao Tong University, Shanghai 200240, China.; ^6^ School of Integrated Circuits, Shanghai Jiao Tong University, Shanghai 200240, China.

## Abstract

Noncontact human-machine interfaces (HMIs) provide a hygienic and intelligent approach for the communication between human and robots. However, they are limited by the interaction distance and bulky power supply. Here, we introduce a self-powered, noncontact intelligent sensing interface based on moisture-driven electricity generation and machine learning technique. We demonstrate that a hydrogel doped with ions exhibits strong hygroelectronic behavior and generates sustainable voltage up to ~0.6 volts from ambient air. The motion of a human hand creates localized air turbulence, resulting in changes to humidity and air pressure that tailor the electrical output. By using machine learning models to decode the motion-dependent voltage, our system achieves high gesture recognition accuracy of up to 99% for Arabic numerals, with an impressive interaction distance up to ~8 centimeters. The proposed system is demonstrated in applications such as encrypted information transmission, virtual reality gaming, and real-time vehicle control.

## Teaser

Hand-induced airflow can induce hygroelectronic signal, enabling self-powered noncontact gesture recognition.

## INTRODUCTION

Human-machine interfaces (HMIs), essential for augmenting the functionality of augmented reality (*1*, *2*) and virtual reality (VR) (*3*, *4*) systems, provide users with various interactive sensations through feedback from hand or fingertip movements (*5*, *6*). These interfaces not only are integral to user-oriented entertainment but also hold potential for applications in surgical robotics (*7*), rehabilitation (*8*), information exchange (*9*), military training (*10*), and beyond (*11*). By enabling users to execute commands through simple hand gestures, HMIs can significantly enhance operational efficiency and user experience.

Current interactive feedback technologies for HMIs are primarily divided into two categories: contact (*12*) and noncontact (*13*–*15*) devices. Contact-based systems, such as those using haptic effects with force feedback (*16*), offer ease of use but suffer from hygiene risks and mechanical wear due to physical interactions. In contrast, noncontact systems that rely on changes in capacitance (*17*, *18*), photonics (*14*, *19*), and thermoelectrics (*20*) bypass physical contact, mitigating these issues. However, for these devices to function effectively over extended periods, a continuous and reliable power supply is essential (*21*, *22*). Traditional battery systems such as lithium-ion batteries, which are typically rigid, bulky, and prone to frequent replacement, present substantial integration challenges and potential safety concerns such as leakage or explosion (*23*, *24*). Sustainable energy harvesting technologies, such as those capturing energy from ambient environmental sources including solar, wind, mechanical energy, have emerged as promising alternatives (*25*, *26*). However, they often face challenges related to device configuration incompatibilities and complex setups (*27*, *28*). Hence, the development of self-powered, energy-harvesting systems with noncontact sensing capabilities is critical, offering greater compatibility, reducing system complexity, minimizing maintenance, and eliminating safety risks (*29*). These systems could harness ambient environmental energy for interactive feedback without the drawbacks of conventional power sources.

Among renewable resources, ambient moisture stands out as a plentiful and recyclable energy source, serving as one of the largest natural energy reservoirs on Earth (*30*–*34*). The ability to generate electricity from moisture, which was first experimentally demonstrated in 2015 has since garnered great attention (*35*). This moisture-driven electricity generation (MEG) technology offers a promising solution for next-generation self-powered electronics (*36*, *37*). By exploiting the interaction between hygroscopic materials and moisture, energy is generated through the movement of free carriers, which can then be harvested and converted into electrical power (*38*). So far, researchers have focused on enhancing electrical output by modifying functional groups to improve water absorption (*37*), increasing ion migration (*39*), and optimizing interfacial properties for charge migration (*40*). These moisture-induced electrical systems have been used to power external devices or serve as self-powered sensors. However, such devices typically produce humidity-dependent direct current (DC) outputs in the presence of consistent water flow (*38*, *41*), posing challenges for sensing and recognizing distinct feedback from various complex commands in HMI applications.

**Fig. 1.**
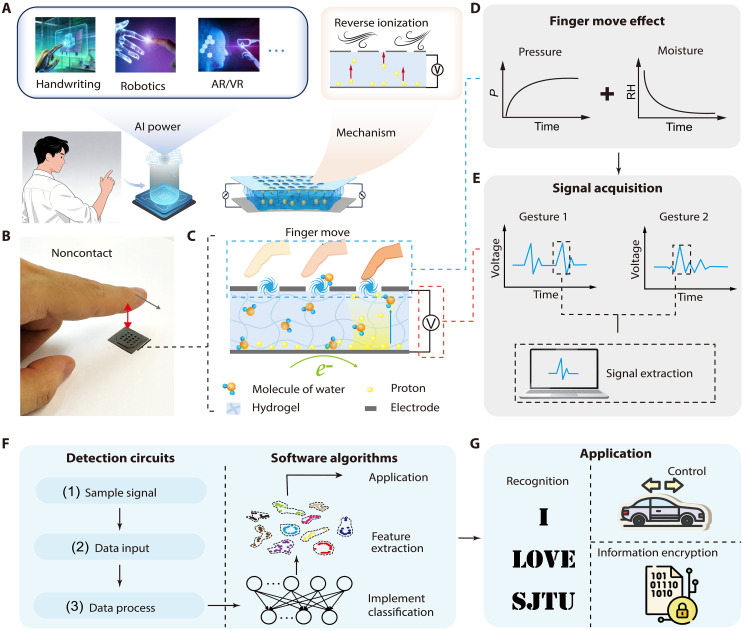
Schematic illustration of the self-powered intelligent sensing interfaces for noncontact gesture recognition system based on turbulence-tailored hygroelectronic effect. (**A**) Schematic of the intelligent sensing system. AI, artificial intelligence; AR, augmented reality. (**B**) Photograph of the intelligent sensing device with noncontact interaction with human finger. (**C**) Schematic of reverse ionization induced by local turbulence caused by finger movement. (**D**) Effects of finger movement on local pressure and humidity of the device over time. (**E**) Schematic of the signal acquisition under different noncontact gesture input. (**F**) Process of detection circuits and classification based on machine learning (ML) algorithms. (**G**) Schematic of the potential applications in recognition, real-time control, and information encryption.

In this study, we present a MEG-based self-powered intelligent sensing interface for noncontact human-machine interactions by using the turbulence-tailored hygroelectronic effect in air (Fig. 1, A and B). By doping a hydrogel with ions, we achieve robust hygroelectronic behavior that generates self-sustained voltage through efficient water absorption and ion migration in porous polymeric networks. When exposed to air, a nearby moving object, such as a human finger, induces localized air turbulence, which is qualitatively defined as localized air turbulence at the hydrogel surface (Fig. 1C). This turbulence leads to a decrease in localized humidity and an increase in air pressure (Fig. 1D), which, in turn, modulates the moisture-device interaction and the generated electrical output (Fig. 1E), with excellent sensing capability under the finger-to-device distances up to 8 cm. The resulting motion-dependent voltage is decoded using machine learning (ML) models (Fig. 1F), achieving an overall recognition accuracy of 99% for Arabic numerals. We demonstrate the device potential in applications such as encrypted information transmission, VR gaming, and remote touchless vehicle control (Fig. 1G). This work paves the way for the development of greener, safer, and smarter HMIs with notable applications in intelligent healthcare, robotics, and transportation.

## RESULTS

### Design principle and fabrication of hygroelectronic device

**Fig. 2.**
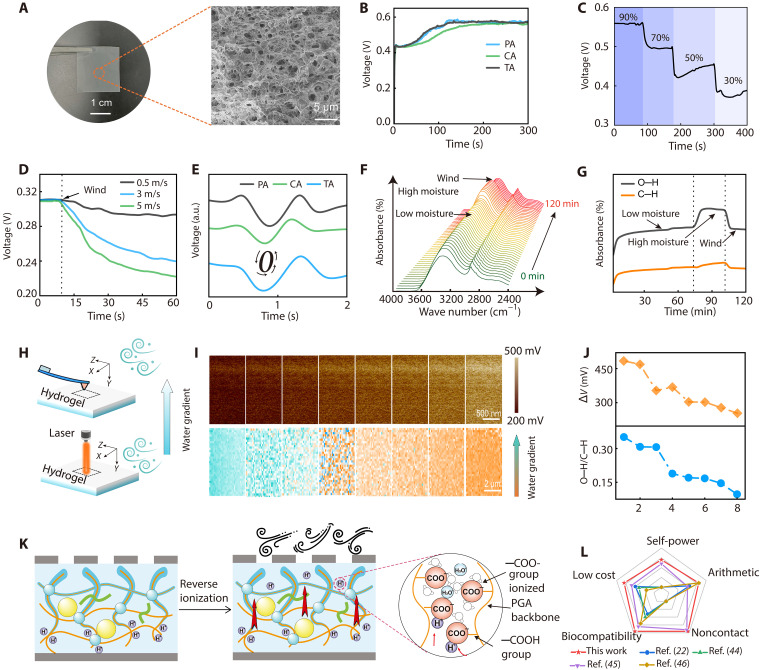
Materials and device characterization. (**A**) Optical image of a representative ionic hydrogel and its corresponding cross-sectional scanning electron microscopy image. (**B**) Output of ionic hydrogel MEG device with different added organic acids of phytic acid (PA), citric acid (CA), and tannic acid (TA) at relative humidity (RH) of 90%. (**C**) Output voltages with different humidity levels. (**D**) Voltage responses under a continuous flow with different speeds at ambient conditions. (**E**) Output of MEG devices with different types organic acid under the handwriting of gestures “0.” a.u., arbitrary units. (**F**) In situ Fourier transform infrared (FTIR) absorbance spectra changing with time (0 to 120 min) in different RH conditions. (**G**) Absorbance intensity of C─H and H─O bonds obtained from (F) under different conditions as a function of time. (**H**) Schematic illustrations of (top) Kelvin probe force microscopy (KPFM) and (bottom) Raman measurement setups under wind conditions. (**I**) (Top row) KPFM characteristics and (bottom row) Raman spectroscopy of the hydrogel film under continuous wind blowing in 1 hour. (**J**) (Top) Surface potential and (bottom) intensity ratio of H─O to C─H bonds, which can be used to indicate the moisture content. (**K**) Schematic illustration of the reverse ionization mechanism under wind. (**L**) Radar chart comparing the key features of the MEG device with other sensing technologies.

To realize a hygroelectronic device capable of generating electricity from ambient moisture, we constructed a hygroscopic ionic hydrogel film by combining polyglycolic acid (PGA), an organic acid, cellulose nanofibers (CNFs), and NaCl (fig. S1). PGA chains form a uniform, robust network with ordered structures under electrostatic attraction from the added metal ions (*42*). This active layer material features abundant micropores (Fig. 2A) and exhibits strong hydrophobicity (fig. S2), which enables it to spontaneously adsorb water molecules from moist air (fig. S3). In the presence of moisture, the numerous carboxyl (─COOH) groups on both PGA and the organic acid (figs. S4 and S5) dissociate, releasing free H^+^ ions through interactions with water molecules (*30*). The relatively large anions (─COO^−^) from the dissociated organic acid remain strongly confined within the PGA network, whereas the much smaller H^+^ ions can move freely, inducing an effective separation of positive and negative charges. Additionally, the incorporated CNF provides extra channels for ion migration in MEG. As a result, driven by an ion concentration gradient, the purely diffusive motion of these ions across the active layer generates an electric potential and an associated current flow (Fig. 2B).

After optimizing key parameters, including the film thickness (~80 μm; fig. S6), the metal cation species (Na^+^; fig. S7), the temperature (25°C; fig. S8), and the electrode geometry (fig. S9), the MEG device sandwiched between carbon electrodes can sustainably deliver an open-circuit voltage of ~0.55 V in a humid environment (Fig. 2B and fig. S10). This output is very robust, regardless of which organic acid is used in MEG. In addition, the sign of the output voltage reverses upon swapping the electrode connections (fig. S11), confirming that the voltage is generated by the device. Because the electricity generation process is strongly tied to water absorption, the device output is highly dependent on ambient humidity. The output is gradually disappeared when the device was placed in a vacuum chamber (fig. S12). As shown in Fig. 2C, the open-circuit voltage increases from ~0.38 V at 30% relative humidity (RH) to ~0.58 V at 90% RH. In addition, the device also shows stable output during multiple charge-discharge cycling experiments (fig. S12).

Notably, the device output is also highly sensitive to airflow in its surroundings (fig. S13). As illustrated in Fig. 2D, under ambient condition (25° ± 5°C) with RH maintained at 30%, the output voltage drops from ~0.3 to ~0.22 V within 50 s when an airflow of 5 m/s is applied to the device. Even a gentle airflow of just 1 m/s can induce a significant change in the output voltage, demonstrating excellent tunability of the voltage output by modulating the local airflow. Thus, this tunable hygroelectronic effect can be harnessed for detecting noncontact handwriting as shown in Fig. 2E. When a human finger writes a “0” in the air at a distance of ~2 to 8 cm above the device, the device produces a characteristic signal waveform with two peaks and two valleys. Impressively, this noncontact handwriting–induced signal is highly reproducible across different devices, regardless of the organic acid used (Fig. 2E), suggesting that the platform can reliably sense finger motion. In addition, different gestures produce distinguishable responses. For example, writing a “1” yields a signal pattern with a single peak and single valley (figs. S14 and S15). This response is maintained even at finger-to-device distances up to 8 cm (fig. S14) and across a wide range of RHs (30 to 70%; fig. S15), indicating reliable performance for noncontact sensing (fig. S16).

### Mechanisms of turbulence-tailored hygroelectronic effect

To investigate how local airflow affects the hygroscopic material and its electrical output, we performed in situ Fourier transform infrared (FTIR) spectroscopy, Kelvin probe force microscopy (KPFM), and Raman spectroscopy on the hydrogel. The FTIR spectrum (Fig. 2F) shows clear peaks corresponding to C─H (1750 cm^−1^) and O─H (3250 cm^−1^) bonds (*30*), arising from the polymer backbone and absorbed water, respectively. The changes of two peaks during applying moisture and wind are summarized in Fig. 2G. The C─H peak remains essentially unchanged, as expected for the stable polymer chains. At low RH, both absorbance bands increase gradually. Upon raising the RH, the O─H absorbance rises sharply, indicating rapid water uptake. However, when a steady airflow is applied to the film, the O─H peak intensity quickly drops, suggesting significant dehydration of the hydrogel network even at high RH. This dehydration is further confirmed by in situ Raman spectroscopy (Fig. 2H, bottom). In a moist environment, the Raman spectrum shows two peaks at ~3300 and ~2950 cm^−1^, corresponding to O─H and C─H bonds (*42*), respectively (fig. S17). We mapped the water content on the hydrogel surface via spatially resolved Raman mapping, using the intensity ratio of the O─H band to the C─H band as an indicator (Fig. 2I, bottom; and fig. S17). The color change in the map, along with a drop in this ratio from ~0.32 to ~0.12 (Fig. 2J, bottom), indicates that dehydration occurs under airflow.

We also used KPFM to examine how airflow-induced dehydration influences the surface potential of the hydrogel (Fig. 2H, top). The top surface of the hydrogel is initially negatively charged because of the migration of dissociated positive ions from the top to the bottom (*43*). Upon applying airflow, the absolute value of potential drops from ~480 to 250 mV (Fig. 2, I and J, top; and fig. S18). This decrease suggests reverse ionization and reduced charge accumulation, consistent with the diminished electrical output observed in Fig. 2D. The mechanism of this process is illustrated in Fig. 2K. In essence, airflow causes reverse ionization that modulates the internal electrostatic field, thereby altering the output voltage signal. This effect can be leveraged for self-powered, noncontact sensing. Figure 2L compares our system with previously reported gesture recognition platforms (*22*, *44**–**46*), highlighting advantages such as self-powering, low cost, biocompatibility, noncontact operation, and easy algorithmic integration.

**Fig. 3.**
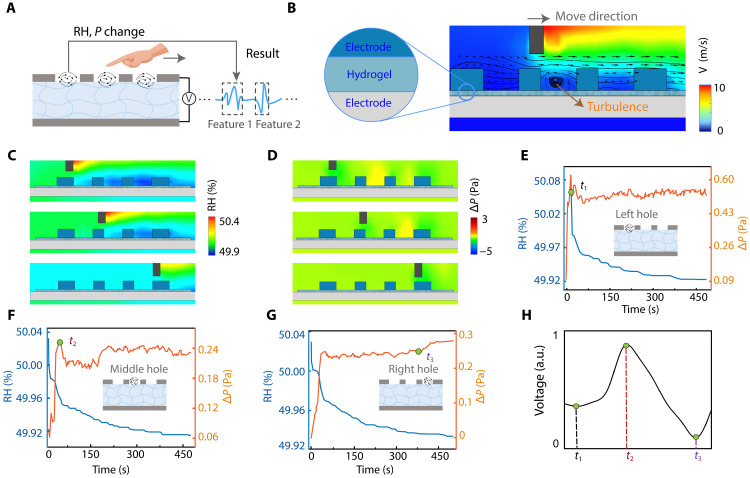
Investigation of turbulence-tailored hygroelectronic effect. (**A**) Schematic of the finger-modulated output by changing humidity and pressure from localized turbulence. (**B**) Flow simulation field maps of the velocity around the device during the rod moving from left to right to mimic gesture “1” input with a distance. Flow simulation field maps of the (**C**) RH and (**D**) Δ*P* around the device during the rod moving at different stages. Local RH and Δ*P* at the (**E**) left, (**F**) middle, and (**G**) right hole positions. (**H**) Illustration of the actual waveform of device output with different times corresponding in (E) to (G). a.u., arbitrary units.

To further elucidate the dynamic airflow conditions during actual noncontact handwriting (Fig. 3A), we modeled the localized flow field around the device (Fig. 3B). The simulation uses device dimensions based on the real devices (see Materials and Methods). In the model, a rod moves above the device from left to right to mimic a finger writing the gesture “1.” To highlight the effect of motion, the finger movement velocity was intentionally amplified in the simulations. While the actual movement speed is ~1 to 2 m/s, a velocity of 10 m/s was used in the simulation. The moving rod induces a strong airflow above the device, with peak velocities reaching ~10 m/s on the right side of the rod. Across most regions including within the mesh electrode holes, the airflow is directed uniformly to the right. However, pronounced turbulence appears in the mesh hole directly beneath the rod (Fig. 3B and fig. S19), indicating intense air fluctuations due to the confined geometry defined by rod and electrode mesh hole. This airflow pattern has two competing effects on the hydrogel. On one hand, the enhanced airflow causes rapid dehydration, leading to a gradual decrease in local RH (Fig. 3C), which reduced humidity tends to diminish the electrical output. On the other hand, the localized turbulence slightly increases the air pressure on the hydrogel surface, which promotes charge migration and thus tends to enhance the output (fig. S20). These opposing effects occur simultaneously, resulting in a distinctive net voltage output that reflects the finger motions. Thus, this turbulence-tailored output enables the self-powered, noncontact sensing illustrated conceptually in Fig. 3A.

Next, we examine how localized turbulences in different electrode holes shape the overall output waveform. Detailed profiles of RH and pressure changes in three representative mesh holes are shown in Fig. 3 (E to G) and fig. S21. In all regions, the local RH decreases slightly (e.g., from ~50.08 to ~49.92%), while the change of local pressure (Δ*P*) increases by more than 0.25 Pa as the finger passes. In the left hole (Fig. 3E), which is affected first by the airflow, both RH and Δ*P* rise sharply at the onset of the motion. The pressure perturbation then stabilizes at time *t*_1_, after which the RH change becomes slow. Consequently, for *t* < *t*_1_, the output voltage shows only a small deviation (Fig. 3H). However, in the middle hole (Fig. 3F), the pressure takes longer to stabilize, reaching steady state at *t*_2_ > *t*_1_. During the interval *t*_1_ < *t* < *t*_2_, Δ*P* is still increasing sharply, while the RH change has begun to level off. The output is therefore dominated by the pressure rise in this period, leading to an increase in voltage (Fig. 3H). The right hole (Fig. 3G) shows a similar trend, except that Δ*P* exhibits a slight secondary increase after *t*_3_. For *t*_2_ < *t* < *t*_3_, Δ*P* has plateaued, while RH continues to drop, so the output is dominated by the humidity decrease and the voltage correspondingly dips (Fig. 3H). Last, for *t* > *t*_3_, RH stabilizes, and a small late increase in pressure causes a final uptick in the output voltage. These time-resolved results illustrate how the combined turbulence effects produce a complex but deterministic voltage waveform in response to a noncontact gesture.

### Neural networks and recognition

**Fig. 4.**
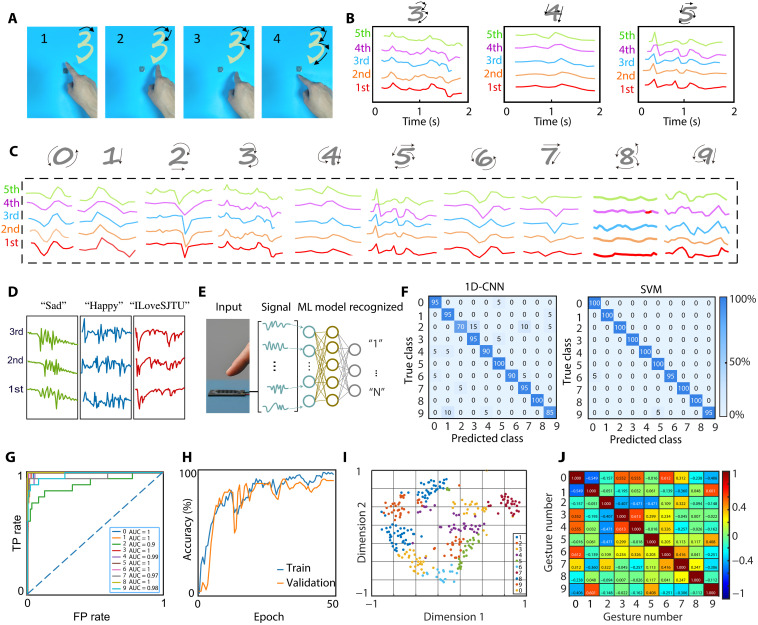
Neural networks and noncontact recognition. (**A**) Photograph of handwriting process with input of numeral “3.” (**B**) Device output recorded with five channels under the handwritings of numerals “3,” “4,” and “5.” Black arrows indicate the stroke orders. (**C**) Signals and stroke sequences for handwritten of numerals “0” to “9.” (**D**) Signals for the handwritten of words “Sad,” “Happy,” and the phrase “ILoveSJTU.” (**E**) ML model architecture of one-dimensional convolutional neural network (1D-CNN) for gesture recognition. (**F**) Confusion matrix of the classification results with the recognition of 10 numerals using (left) 1D-CNN and (right) SVM models. (**G**) Receiver operating characteristic and (**H**) training/validation learning curves for the ML algorithm using 1D-CNN. AUC, area under the curve. TP, true positive; FP, false positive. (**I**) Clustering scatter plot for the 10 numerals, indicating the minimum overlap. (**J**) Correlation-matrix heatmap across the 10 numerals.

The above analysis demonstrates that the turbulence-tailored hygroelectronic effect produces output signals that closely mirror the finger motion. Notably, the signal patterns remain robust across different finger distances and ambient humidity levels (figs. S14 and S15). Therefore, changing the handwriting gesture leads to a corresponding change in the output voltage waveform (Fig. 4). Gesture recognition plays an essential role in HMIs, yet many existing systems rely on bulky power supplies and require direct contact, leading to wear and fatigue. By contrast, our turbulence-tailored hygroelectronic interface is self-powered and contact free, representing a step toward more efficient, convenient, and intelligent HMI devices. The finger motion itself generates the sensing signal, eliminating any need for an external power source and avoiding direct physical contact. This approach addresses key shortcomings of traditional handwriting recognition devices in HMI.

Our contactless sensing interface can accurately capture each handwritten stroke in real time (Fig. 4, A to C). For example, Fig. 4A shows the discrete finger trajectory when writing the numeral “3” over a ~2-s interval. Using a five-channel setup, the finger motion produces distinct voltage waveforms in each channel (Fig. 4B). Writing the numeral “3” consists of two repeated strokes, and, accordingly, our sensor outputs two similar waveform patterns. The device similarly captures each stroke when writing numerals “4” and “5” with high fidelity. Figure 4C compiles the voltage signals for handwritten numerals 0 through 9, demonstrating that each digit yields a characteristic waveform signature. Moreover, beyond Arabic numerals, the exceptional sensitivity of our device also enables effective tracing of letters and words. As shown in Fig. 4D, writing “Sad,” “Happy,” and “ILoveSJTU” generates distinct output waveforms that faithfully track the features of each handwritten word. This outstanding performance greatly broadens the range of recognizable inputs and potential application scenarios.

After signal acquisition, we implemented a one-dimensional convolutional neural network (1D-CNN) to classify the sensor outputs (Fig. 4E). Compared to recurrent models, 1D-CNNs can capture local motifs as well as long-range dependencies through hierarchical receptive fields, providing a simple and efficient solution for sequence classification with relatively small datasets (*22*). We optimized the CNN architecture by tuning the convolution kernel size and the number of filters (fig. S22). The best performance was achieved with a kernel size of 7 and 72 filters (using a batch size of 128). With only ~20 training samples per class, the optimized model achieved overall recognition accuracy of 91.5% for these digits (Fig. 4F, left; and fig. S23). Notably, with even simpler classification model of support vector machine (SVM) model can also attain high accuracy of 99% with our dataset (Fig. 4F, right). The receiver operating characteristic curves (Fig. 4G) indicate an area under the curve of ~1.0, highlighting the excellent discrimination of the classification algorithm. The training and validation learning curves converge smoothly with negligible overfitting (Fig. 4H), reflecting effective regularization and adequate data diversity. To visualize how the network distinguishes different inputs, we extracted the learned feature vectors from the latent space and projected them into two dimensions. The resulting plot (Fig. 4I) shows well-separated clusters for all 10 numerals with minimal overlap, illustrating the strong feature extraction and clustering capabilities.

We further analyzed the sensor signals via correlation analysis to quantify the distinctions between different handwritten inputs (Fig. 4J). For example, the correlation coefficient between the outputs for “0” and “6” is about 0.612, reflecting the similarity of their circular strokes. Across all numeral pairs, only ~6% exhibits a correlation higher than 0.6, underscoring the excellent discriminability of the recognition system. Multiview correlation analyses (Pearson, Spearman, and first difference; fig. S24) confirm that the CNN-learned features are robust to minor variations while remaining sensitive to unique dynamics of each numeral strokes. Overall, our system achieves 91.5% recognition accuracy with a recall rate of ~100% (fig. S25). Notably, a conventional random forest model can also attain high accuracy with our dataset (fig. S26), highlighting the reliability of the turbulence-tailored hygroelectronic signals. Collectively, these results establish a complete pipeline from self-powered signal acquisition to intelligent inference, with interpretability supported by the signals’ correlation structure and the clear clustering geometry of the feature space.

### Application of self-powered, intelligent, and contactless sensing interfaces

Last, we demonstrate applications of our self-powered, noncontact sensing interface in secure cryptographic message transmission and real-time remote control. Encrypted communications (for instance, in banking, online transactions, or military scenarios) traditionally rely on physical input of keys or passwords, which can suffer from a lack of concealment, susceptibility to eavesdropping, and the need for powered hardware (*47*). Our noncontact interface addresses these issues by allowing encryption keys to be entered invisibly through hand gestures, without any physical connection or external power.

**Fig. 5.**
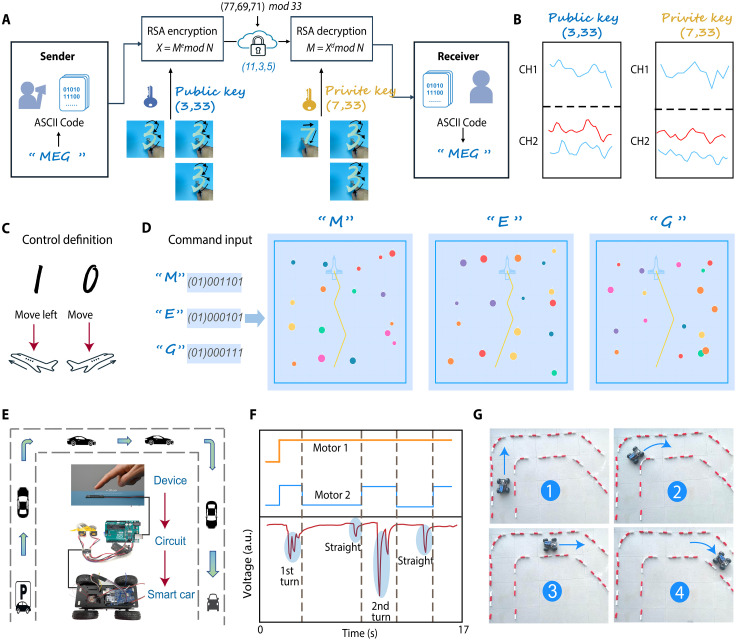
Demonstrations of secure cryptographic message transmission and real-time remote control based on self-powered intelligent sensing device in contactless mode. (**A**) Schematic of a workflow in which signals from a MEG are fed to a trained ML model and used in proof-of-concept RSA encryption application. A single representative image is used to illustrate the hand gesture inputs corresponding to different numbers. (**B**) Raw signal waveforms corresponding to the RSA public and private keys. (**C**) Definition of command for controlling airplane movement in arcade-style video game. (**D**) Demonstration of the control of airplane to avoid obstacles under the command sequence recognition of received “MEG” message at the end user. (**E**) Schematic of the real-time control of smart car under the interaction with human finger recognized by the device. (**F**) Motor-control logic and measured voltage signals from MEG device for command recognition during smart car operations of going straight and turning right. a.u., arbitrary units. (**G**) Images of the smart car in operation with tasks of two turns to reach destination.

We developed a proof-of-concept encrypted transmission using our device (Fig. 5A). The message “MEG” was converted into ASCII code and then encrypted into ciphertext (11, 3, 5) using Rivest-Shamir-Adleman (RSA) encryption, with a public key (3, 33) provided via a contactless handwritten gesture captured by the device. The two-channel output for this gesture (Fig. 5B) was processed by the classifier to yield the digits of the public key. On the receiving side, the ciphertext was decrypted using the corresponding private key (7, 33), which was likewise entered through a noncontact gesture and recognized by the device (Fig. 5B). The original message “MEG” was successfully recovered after decryption (Fig. 5A). By entering both the public and private keys through contact-free gestures, the key input is physically concealed, greatly enhancing security.

Encrypted messages transmitted by our system can also trigger actions in a remote end user. We created a small arcade-style video game to demonstrate this capability (Fig. 5C). In the game, the digits “0” and “1” recognized by our device are mapped to the commands “move right” and “move left,” respectively. We then used the decoded letters of “MEG” as a sequence of game commands for an avatar. For example, the letter “M” was encoded as the command sequence (01)001101, where the prefix “01” acts as a start trigger (Fig. 5D). This binary sequence translates to the moves: right, right, left, left, left, right, left. When these commands were sent to the game, the avatar (an airplane) followed them precisely, successfully avoiding obstacles as shown in Fig. 5D. The command sequences corresponding to “E” and “G” were executed similarly and achieved high accuracy in navigation (movie S1). This gaming demonstration highlights the high recognition accuracy and secure information transmission enabled by our system.

Beyond virtual environments, we also integrated our sensor with a hardware platform to achieve real-time remote control of a physical device. As illustrated in Fig. 5E, the sensor was connected to a microcontroller board running our classification algorithm, and this board, in turn, controlled a two-wheeled smart car (figs. S27 and S28 and movie S2). The car moves straight when both motors run in sync, and it turns when only one motor is powered. In one trial (Fig. 5F), the user wrote a single stroke “1” in the air. The sensor generated a voltage waveform with one prominent peak, which the system recognized as the “go straight” command. The microcontroller then activated both motors, causing the car to drive straight forward (Fig. 5G). In another trial, the user wrote a double-stroke “11,” producing a waveform with two distinct peaks. The system interpreted this as a “turn right” command and accordingly drove only the left motor, causing the car to pivot to the right (Fig. 5G). In a full demonstration, we tasked the car to navigate a track with two turns (Fig. 5G). Using a sequence of noncontact handwritten commands over ~17 s, the car successfully executed two turns and reached the destination (movie S3). These results showcase the potential of our MEG-based sensing interface for both secure communication and real-time control scenarios, pointing to an innovative paradigm for intelligent systems that are contactless and battery free.

## DISCUSSION

In summary, we have developed a self-powered, green energy-harvesting, and intelligent sensing platform based on a turbulence-tailored hygroelectronic effect in air. This system offers substantial potential for intelligent human-machine interactions, including noncontact motion recognition, secure information transmission, and real-time remote control. The core of the platform is a doped hydrogel that efficiently generates a self-sustained voltage up to ~0.6 V through continuous water absorption and ion migration in a porous polymer network. Irregular finger motions create localized air turbulence at the hydrogel surface, which induces simultaneous fluctuations in local humidity and air pressure. These fluctuations dynamically modulate the MEG electrical output, with excellent sensing capability under the finger-to-device distances up to ~8 cm. We then decode the output voltage into gesture information using ML models, achieving recognition accuracy up to 99% for Arabic numerals. The integration of these components enables a wide range of applications, such as high-accuracy noncontact handwriting recognition, contact-free encryption/decryption, and remote directional control in both virtual and real environments. Although some environmental factors such as background airflow may have interference to the signal output, enhanced signal processing algorithms can be added to exclude these possible interferences for practical deployment. Collectively, the combination of self-powering, noncontact sensing, and intelligent signal processing establishes a previously unknown paradigm for battery-free, human-interactive electronic devices.

## MATERIALS AND METHODS

### Fabrication of MEG film

PGA (5 g) was dissolved in 10 ml of deionized water under continuous magnetic stirring at room temperature until a homogeneous solution was achieved. A 10% (w/v) organic acid solution (e.g., tannic acid, citric acid, or phytic acid) was prepared by adding 2 ml to the PGA solution, followed by stirring for 3 min. Subsequently, 0.1 g of sodium chloride (NaCl) was introduced and stirred until fully dissolved. CNFs were then incorporated into the mixture, and stirring continued for an additional 5 min. The resulting formulation was drop-casted onto a carbon electrode to form the lower-electrode/intermediate-layer film. The coated substrate was placed in an oven at 60°C for 12 hours to facilitate complete drying. The film thickness was controlled by the dispensed volume and the duration of the drying process.

### Fabrication of MEG device

The MEG device was fabricated with a sandwich architecture comprising a bottom carbon electrode, a hygroscopic intermediate layer, and a top carbon electrode, which was patterned with a 3 by 3 array of 2-mm perforations. The layers were sequentially stacked, ensuring optimal alignment and gentle lamination to achieve intimate interfacial contact, resulting in a fully assembled MEG device. Electrical interfacing was achieved by bonding lead wires to the top and bottom carbon electrodes using conductive adhesive. The leads were then connected to a source meter or a microcontroller acquisition board, with the detection circuit enabled for reliable transmission of sensor signals.

### Material characterization

Surface potential measurements were carried out by KPFM using a Multimode 8 atomic force microscopy system (Bruker). The hydrophobicity of the hydrogel films was evaluated by static water contact angle measurements using a DSA30 contact angle analyzer (KRUSS GmbH). Optical images of the samples were acquired using an Olympus BX53 optical microscope. The microstructure and morphology of the films were examined by field-emission scanning electron microscopy (Thermo Fisher Scientific Apreo 2S) operated at an accelerating voltage of 5 kV. A continuous air flow was supplied using an adjustable blowing machine to provide a controlled airflow environment during measurement. Chemical and bonding information was obtained using Fourier-transform infrared spectroscopy (PerkinElmer Spectrum 100) equipped with an attenuated total reflection accessory. Elemental composition and chemical states were further analyzed by x-ray photoelectron spectroscopy (ESCALAB Xi+). Spatially resolved water absorption and dehydration kinetics were probed by confocal Raman mapping (Renishaw inVia Qontor, λ = 532 nm). Raman measurements were performed under controlled RH conditions to monitor changes in vibrational modes and to construct spatial maps of moisture redistribution at the hydrogel surface.

### Flow simulation settings

The computational domain for the airflow simulation was defined as a cubic space of 10 m by 10 m by 10 m. The air was modeled under standard atmospheric conditions, with material parameters for the carbon electrodes following standard carbon-paper data. The hydrogel was represented using the primary composition of PGA, and the upper and lower electrodes were modeled as 1-mm-thick plates. The air region was discretized using a base mesh size scaled to the domain, while the solid subdomains of electrodes and hydrogel were meshed with a nominal element size of 0.1 mm. The initial ambient temperature and humidity were set at 25°C and 30%, respectively. To simulate human interaction, the prescribed “finger motion” was set to a velocity of 10 m/s. All remaining numerical settings and constants are provided in table S1.

### Signal clustering methodology

The raw sensor signals were preprocessed for clustering analysis by resampling the data to a fixed length of 30 points per trace and normalizing the signals on a per-sample basis to ensure metric comparability. Unsupervised clustering was performed using the *k*-means algorithm with Euclidean distance as the objective function (*k* = 10) and multiple replicates to mitigate initialization bias. Cluster assignments were then evaluated in the original feature space. To facilitate interpretability, the high-dimensional samples were embedded into a two-dimensional plane using *t*-distributed stochastic neighbor embedding (*t*-SNE). This dimensionality reduction technique was used solely for visualizing the separation of clusters and understanding neighborhood structures. Last, the unsupervised clustering results were compared to ground-truth digit classes via a confusion matrix to qualitatively assess clustering consistency.

### 1D-CNN and other ML training

For gesture recognition, we used a 1D-CNN designed to process three-channel inputs derived from each resampled waveform (length of 320). The input channels consisted of the *z*-scored signal, its first-order difference, and a log-magnitude fast Fourier transform channel. Training occurred in two stages: an initial baseline phase using class-balanced cross-entropy with label smoothing, stochastic weight averaging, and an ArcFace classifier, followed by fine-tuning where the backbone was frozen, and a margin-based head with focal regularization was introduced. Pairwise hinge loss was applied to select class pairs. To preserve nontarget classes during fine-tuning, knowledge distillation from the stage A teacher model was used. The mini-batch size was 128, and training was optimized using cosine learning rate warm restarts and an ensemble over multiple random seeds with test-time augmentation.

As classical baselines, we used random forest (200 trees) and a SVM with an RBF kernel, tuned for optimal box constraint and kernel scale. For these models, each signal was resampled to a length of 30 and converted into a compact feature vector that included time-domain statistics (mean, variance, range, root mean square, skewness, kurtosis, and zero-crossing rate) as well as frequency-domain descriptors (dominant frequency/amplitude, spectral centroid, and spectral spread). All features were standardized before training. Evaluation was performed using a fixed test set (20 samples per class) with a separate validation subset drawn from the remaining data.

### Signal correlation analysis

For each numeral class (0 to 9), the first 30 signals were extracted from the raw data and linearly resampled to 33 points to standardize temporal resolution. Class-level prototypes were obtained by averaging the 30 resampled traces, suppressing intraclass noise while preserving the characteristic morphology. Pairwise similarity between classes was computed using Pearson and Spearman correlation methods, producing 10 by 10 correlation matrices. To mitigate slow trends and autocorrelation, similarity was also computed on the first-order differences (Δ*x_t_* = *x_t_* − *x_t_*_−1_). The “Spearman-Pearson” difference maps were generated to highlight discrepancies between linear and rank-based relationships. All correlation matrices were visualized as heatmaps with calibrated axes and fixed color limits, with the mean off-diagonal correlation reported via Fisher *z*-transformation to ensure stability and avoid bias due to self-similarity on the diagonal.

### Microcontroller circuit implementation

Signal acquisition and actuation were coordinated using an Arduino Uno R3, with the device output fed to the board for on-board monitoring. A high-resolution 16-bit analog to digital converter [(ADC)ADS1115] was used for signal readout. A passive resistor-capacitor (RC) low-pass filter (cutoff of ~50 to 100 Hz, input impedance of ≥100 kΩ) was used to reduce high-frequency noise before the inputs. The ADS1115 operated in single-ended mode with a ±2.048-V full-scale range at a sampling rate of 20 samples per second (SPS). The Arduino Uno was powered through a regulated 5-V USB rail, while the ADS1115 was connected to the 3.3 V regulator of the Uno. All modules shared a common ground. Motors were powered by an isolated 6- to 9-V supply, with the ground star-connected to the logic ground at a single point. The motors were controlled via a dual-channel logic-pulse width modulation (PWM) motor driver, with Arduino PWM signals set to ~1 kHz and duty-cycle updates at 50 to 100 Hz.

## Supplementary Materials

### The PDF file includes:

Supplementary Notes S1 and S2 Figs. S1 to S28 Table S1 Legends for movies S1 to S3
