# Supplementary material for: Moisture-driven, self-powered noncontact sensing interfaces via turbulence-tailored hygroelectronic effect
Source: Sci Adv. 2026 Apr 17;12(16):eaee7050. doi: 10.1126/sciadv.aee7050 (PMC13089349; doi:10.1126/sciadv.aee7050)
Supplement: Supplementary file 1 — Supplementary Notes S1 and S2 Figs. S1 to S28 Table S1 Legends for movies S1 to S3 [file sciadv.aee7050_sm.pdf]

Science Advances
AAAS

Supplementary Materials for

Moisture-driven, self-powered noncontact sensing interfaces via turbulence-tailored hygroelectronic effect

Daozhi Shen *et al.*

Corresponding author: Daozhi Shen, dzshen@sjtu.edu.cn

Sci. Adv. **12**, eaee7050 (2026)
DOI: 10.1126/sciadv.aee7050

The PDF file includes:

Supplementary Notes S1 and S2
Figs. S1 to S28
Table S1
Legends for movies S1 to S3

Other Supplementary Material for this manuscript includes the following:

Movies S1 to S3

Supplementary Notes

Note S1 Details in the fabrication of MEG device

During assembly of the sandwich-structured MEG (carbon lower electrode / PGA–tartaric acid–NaCl–cellulose intermediate layer / perforated carbon upper electrode), the key risks are interfacial adhesion, uniformity of the hygroscopic layer, and low-resistance electrical contacts. The precursor must be fully homogenized before casting (PGA completely dissolved in DI water, tartaric acid added and mixed to uniform pH/ionic strength, NaCl fully dissolved, cellulose dispersed without agglomerates); incomplete mixing leads to local ionic gradients and unstable output. When drop-casting, control the dispensed volume and level the substrate to avoid thickness gradients; gently debubble the slurry to prevent pinholes beneath the 3×3 array of 2-mm perforations. After coating, dry the film in a drying oven for 12 h without forced airflow directed at the surface to minimize coffee-ring effects and edge cracking; avoid over-drying that embrittles the hydrogel. Align the perforated upper electrode accurately to the coated area to ensure repeatable vapor pathways and to prevent shorting at the edges; Electrical leads should be bonded to both carbon electrodes using conductive adhesive with minimal bondline thickness; To limit corrosion or contamination from salts, keep metal fixtures away from exposed electrolyte during assembly and clean any residue from electrode edges before encapsulation or wiring. Perform a quick pre-integration check—visual inspection for voids, sheet-resistance mapping of electrodes, and an open-circuit voltage stability test under ambient humidity—before connecting the device to the source meter or microcontroller input. Environmental control during fabrication (stable room temperature, moderate RH) and gentle handling of the perforated top electrode further improve yield and device-to-device consistency.

Note S2 Details of model data processing

We use a lightweight 1D-CNN with a two-stage ("surgical") schedule to keep the model compact while selectively improving hard classes. We first train the full network with class-balanced and label smoothed cross-entropy. We label smoothing distributes a small mass ε over non-target classes, and class weights are normalized to unit mean to stabilize optimization.

$$L_{CE} = -\left(\frac{1}{B}\right) * sum_{\{i=1..B\}} sum_{\{c=1..K\}}\left(\varpi_c * q_{i(c)} * \log\left(softmax(z_i)_c\right)\right) \quad \text{(S1)}$$

The classifier head uses ArcFace with class-dependent margins to tighten decision boundaries (stronger margins may be assigned to target classes that are frequently confused), where $\theta_{\{i,c\}}$ is the angle between the normalized feature and class weight.

$$z_{i(y_i)} = s * \cos\left(\theta_{\{i,y_i\}} + m_{\{y_i\}}\right),\ z_{i(c)} = s * \cos\left(\theta_{\{i,c\}}\right)\ (c\ != y_i) \quad \text{(S2)}$$

We then freeze the backbone and updates only the head, combining the same CE with a light pairwise hinge on specified confusing class pairs and knowledge distillation from the teacher from first stage to protect non-target classes. This concentrates capacity on targeted fixes without regressing others.

$$L_B = L_{CE} + lambda_{KD} * T^2 * KL\left(softmax\left(\frac{t}{T}\right) \parallel softmax\left(\frac{z}{T}\right)\right) + \alpha * L_{pair} \quad \text{(S3)}$$

At inference, simple test-time averaging of lightly augmented traces stabilizes predictions; overall, this design yields strong accuracy with minimal parameters and maintains robustness on non-target classes.

Supplementary Figures

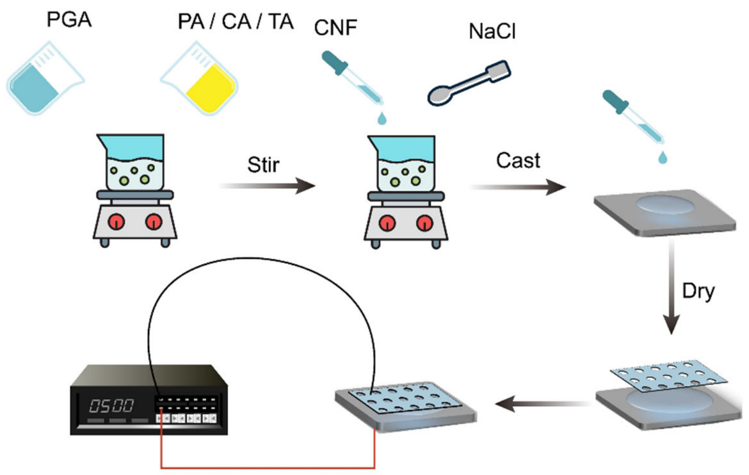

Fig. S1 Fabrication process and electrical measurement of MEG devices.

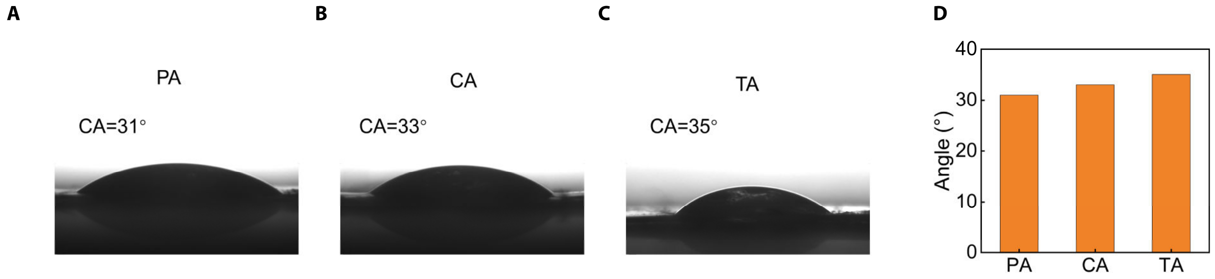

Fig. S2 Water contact angle measurement of active materials with different added organic acids. (**A**) PA. (**B**) CA. (**C**) TA. (**D**) Contact angles summary.

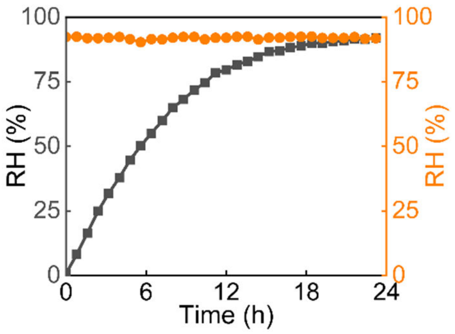

Fig. S3 Change of hydrogel mass with time in a moist environment at 90% RH.

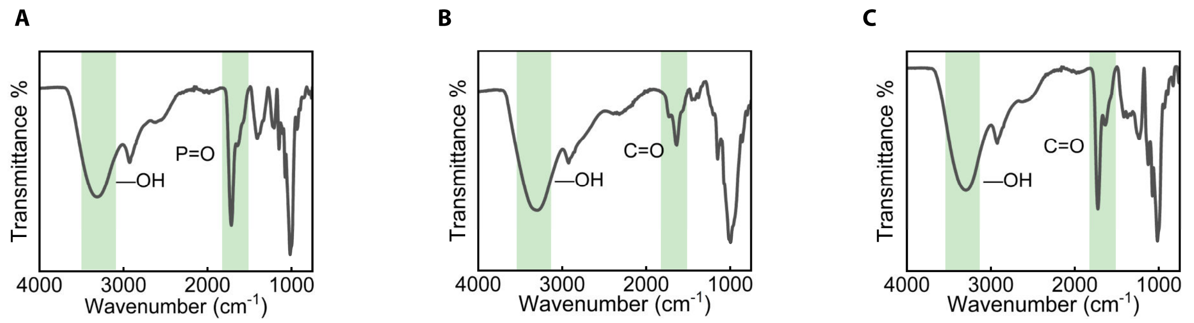

Fig. S4 FTIR spectra of active materials with different added organic acids. (**A**) PA. (**B**) CA. (**C**) TA.

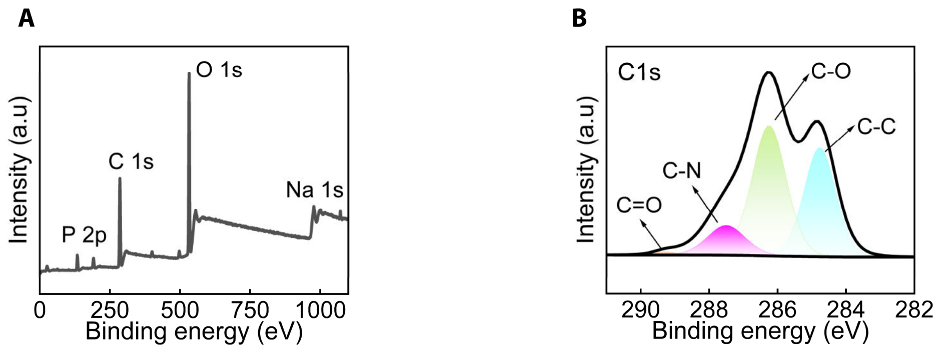

Fig. S5 XPS photoelectron spectra of the hydrogel film. (**A**) XPS full spectrum. (**B**) Intensity of C 1s in the XPS spectrum, where a large number of C-O, C=O bonds, and -COOH groups are present, which significantly enhances the water absorption capacity of the film.

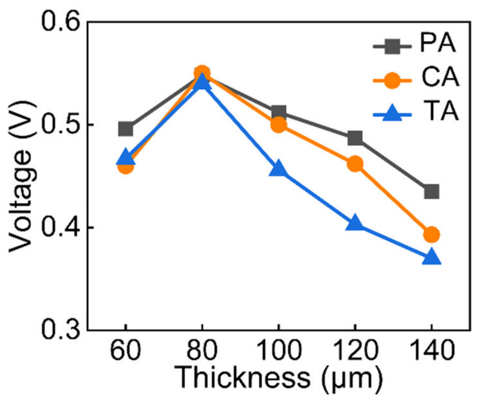

Fig. S6 Output of MEGs with different types of organic acids with different hydrogel thickness at RH of 90% under room temperature.

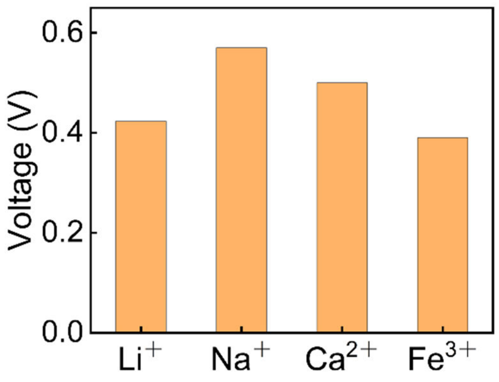

Fig. S7 Output of MEGs with different types of additive metal ions.

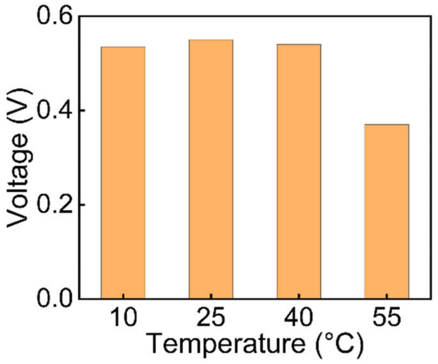

Fig. S8 Output of MEGs under different temperature conditions, with the hydrogel film thickness fixed at 80 μm and humidity maintained at 90%.

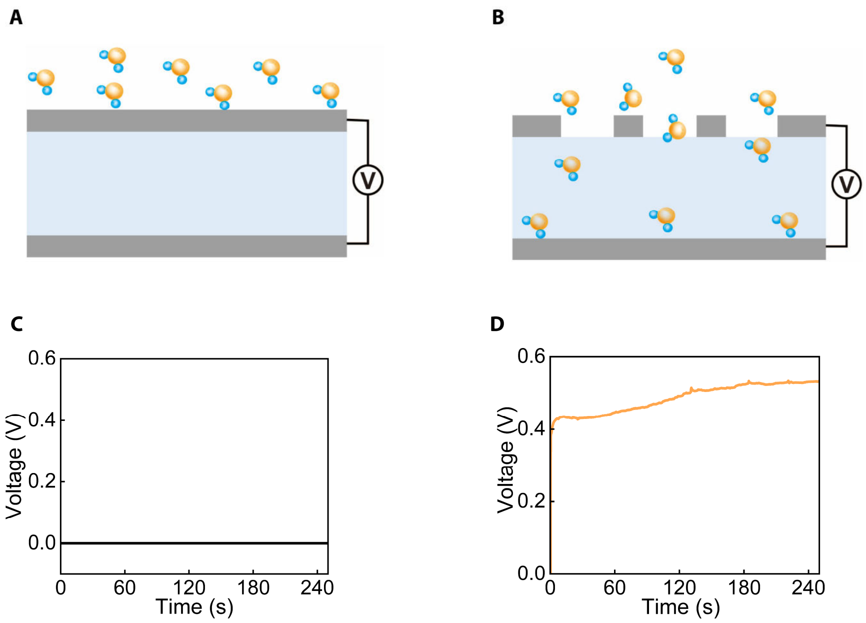

Fig. S9 Output comparison of MEG devices with and without holes in the top electrodes. Schematic diagram of the MEG device with (**A**) no hole and (**B**) meshed holes in the upper electrode. (C) Voltage output curve of the device in (**A**). (**D**) Voltage output curve of the device in (**B**).

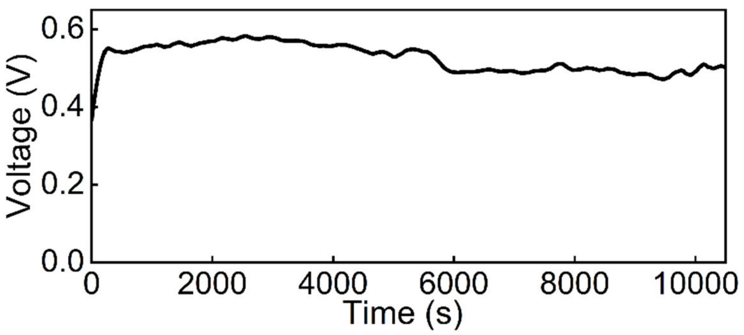

Fig. S10 Long term output characteristic of MEG device in wet environment.

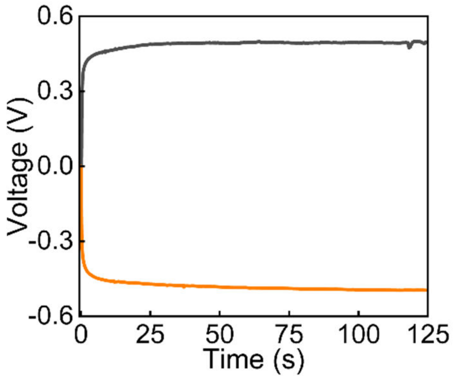

Fig. S11 Output polarity test of MEGs by swapping electrical connections to top and bottom electrodes.

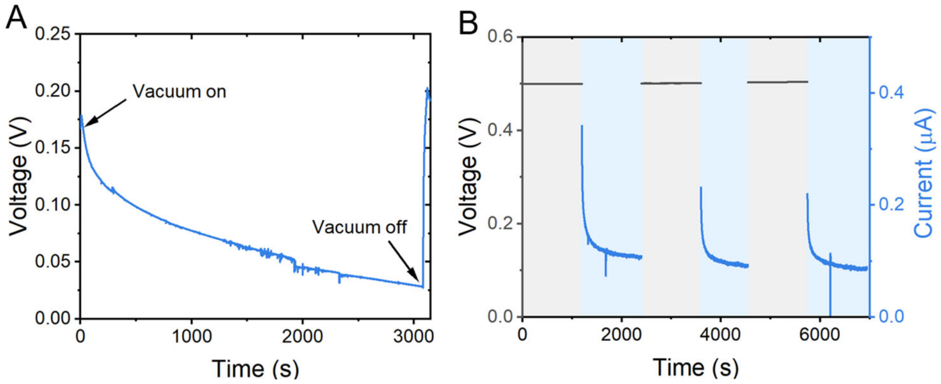

Fig. S12 Device output under different conditions. (A) Device output in cyclic vacuum on and off. (**B**) Device output in cyclic in charging-discharging conditions.

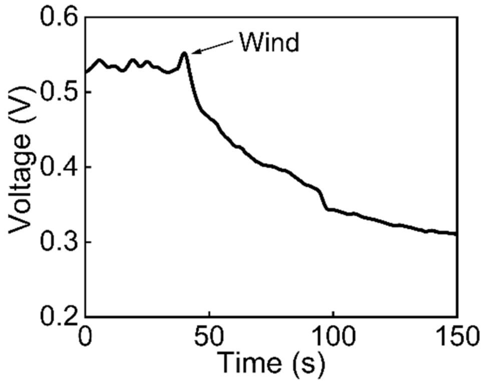

Fig. S13 MEG output upon a continues wind blowing at 5 m/s.

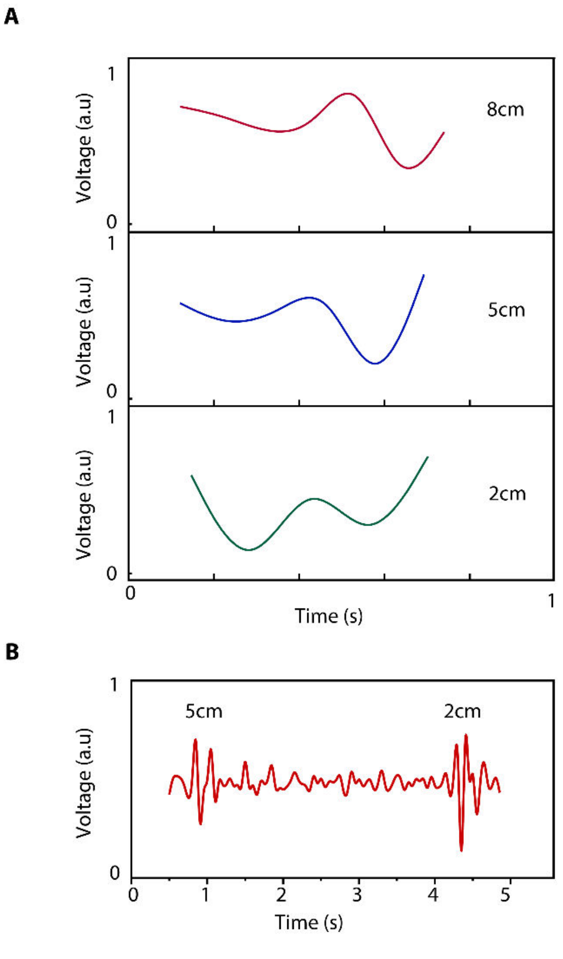

Fig. S14 Output voltage during handwriting of the numeral "1" at different finger-to-device distances. (**A**) Zoom-in views at three distances of 2, 5, and 8 cm. (**B**) Baseline-subtracted raw signals at two different distances.

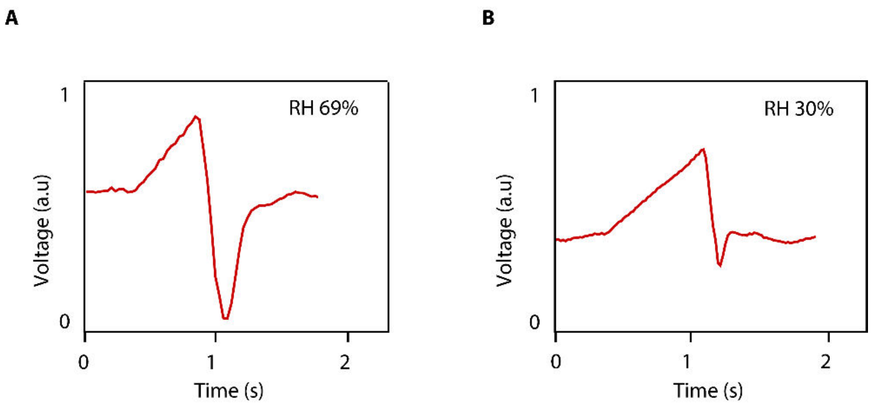

Fig. S15 Output voltage during handwriting of numeral "1" under different humidity conditions. (**A**) RH = 69%. (**B**) RH = 30%.

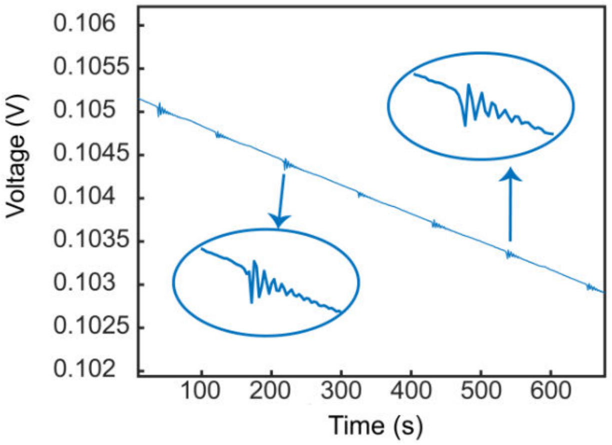

Fig. S16 Continuous segments of the raw voltage signal under repeated handwriting over time.

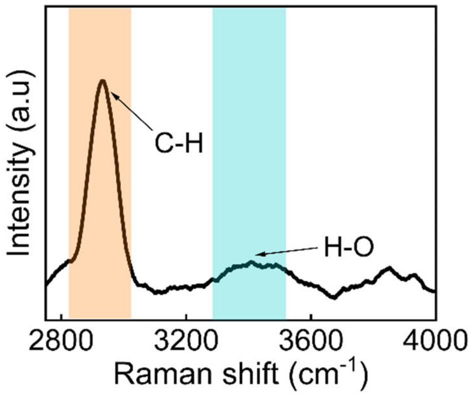

Fig. S17 Raman spectra of the hydrogel film surface, with arrows indicating the integral intensities of the C-H and H-O bonds.

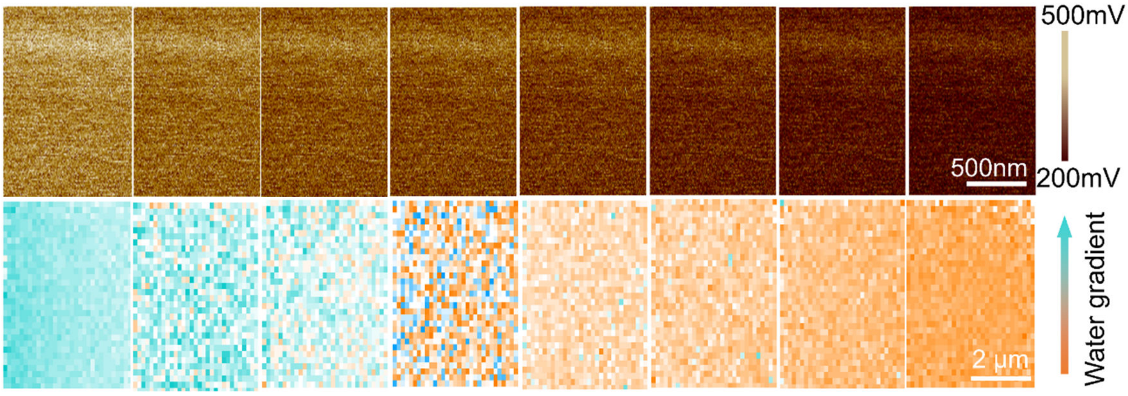

Fig. S18 KPFM characterization of the moisture-active film during wind blowing condition in 1 h. The KPFM bias was set to be 0.5 V.

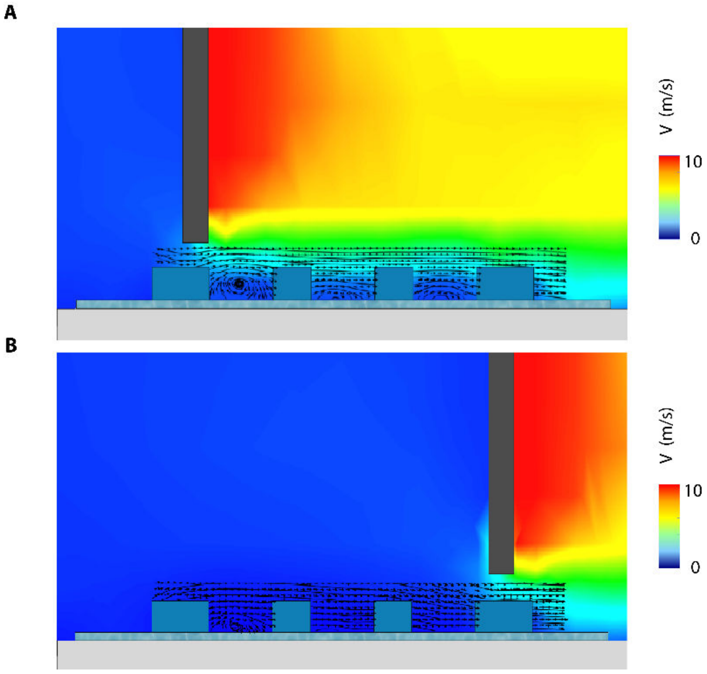

Fig. S19 Flow Simulation results of the velocity field around the device at different stages during finger moving. (**A**) At the onset of the finger swipe. (**B**) At the end of the swipe.

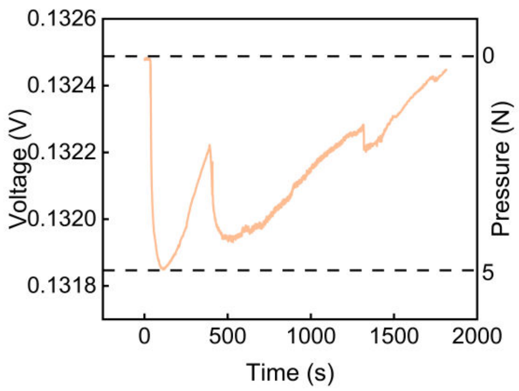

Fig. S20 Effect of external pressure on the MEG output.

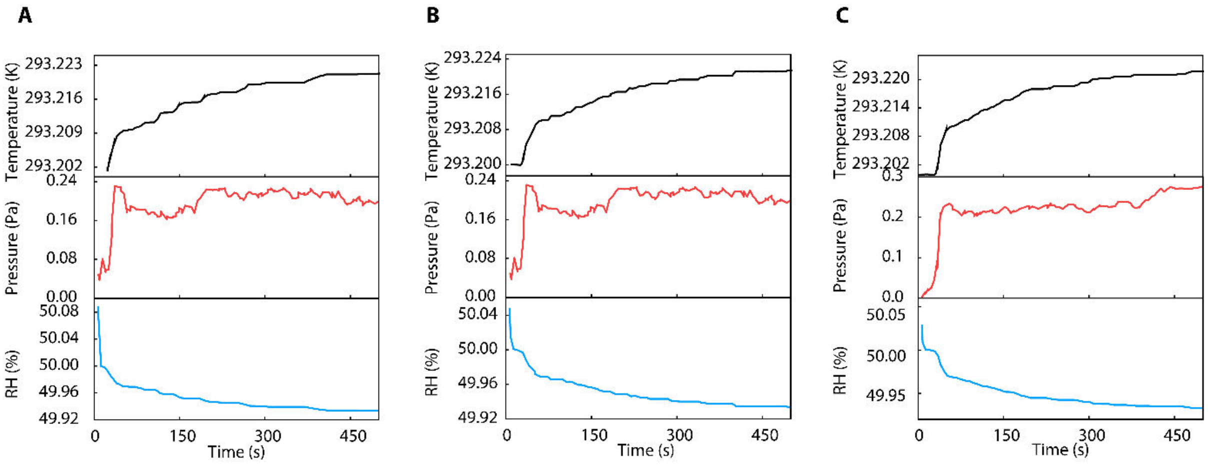

Fig. S21 Simulated time traces of RH, temperature and pressure at different locations. (**A**) First pore. (**B**) Second pore. (**C**) Third pore.

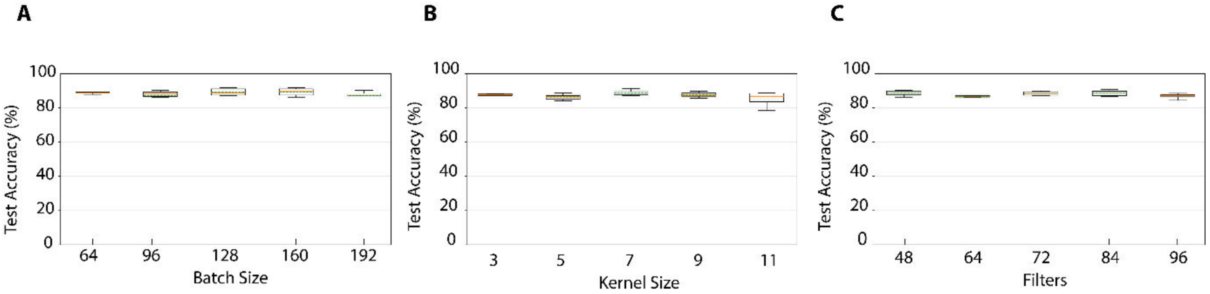

Fig. S22 Hyperparameter-optimization settings for the 1D-CNN model. The obtained test accuracy with different (**A**) batch-size, (**B**) convolutional kernel-size and (**C**) filter settings.

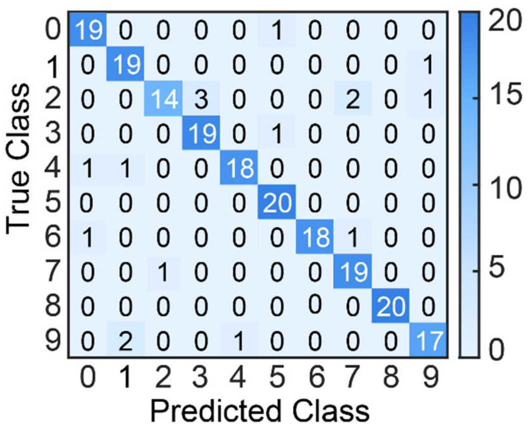

Fig. S23 Confusion matrix for 10 numerals recognition using 1D-CNN model.

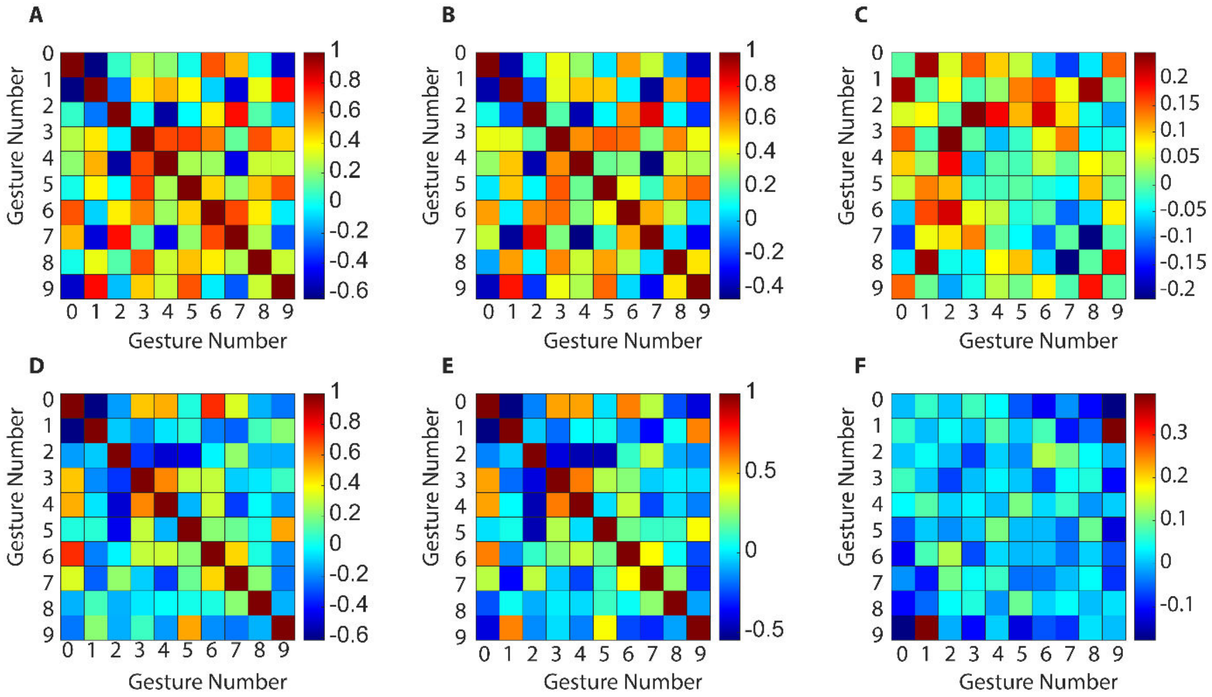

Fig. S24 Correlation analysis across the 10 numerals. (**A**) Pearson correlation-coefficient matrix. (**B**) Spearman rank correlation-coefficient matrix. (**C**) Comparison between Pearson and Spearman. (**D**) Pearson correlation after first-difference preprocessing. (**E**) Spearman correlation after first-difference preprocessing. (**F**) Comparison between the differenced Pearson and Spearman results.

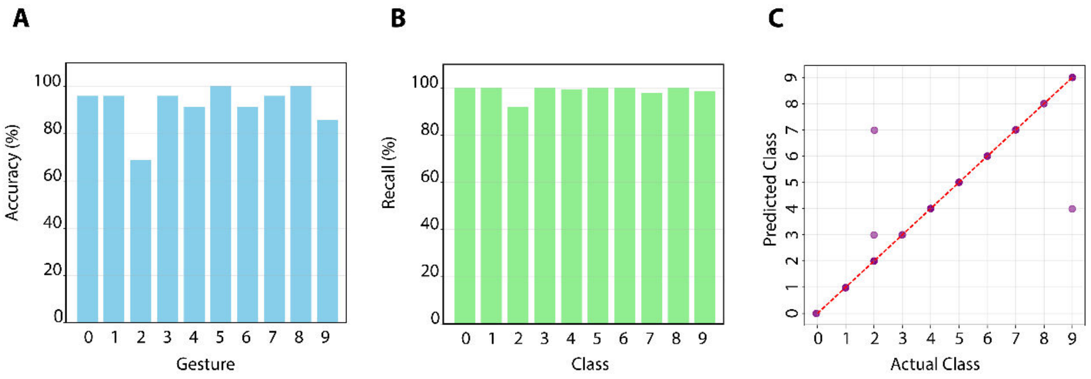

Fig. S25 The evaluation of training of the machine-learning model. (**A**) Accuracy. (**B**) Recall rate. (**C**) Validation scatter plot of numeral predictions.

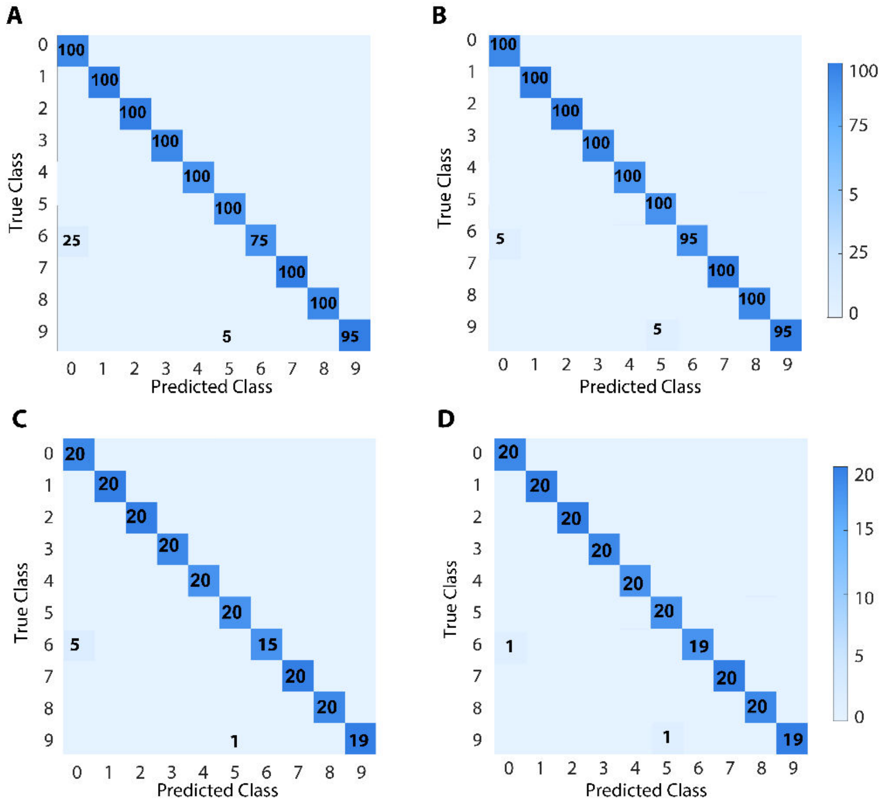

Fig. S26 Classification results and percentage confusion matrices using algorithm of Random Forest and Support Vector Machine. Percentage confusion matrix of (**A**) Random Forest and (**B**) Support Vector Machine. Confusion matrix of (**C**) Random Forest and (**D**) Support Vector Machine.

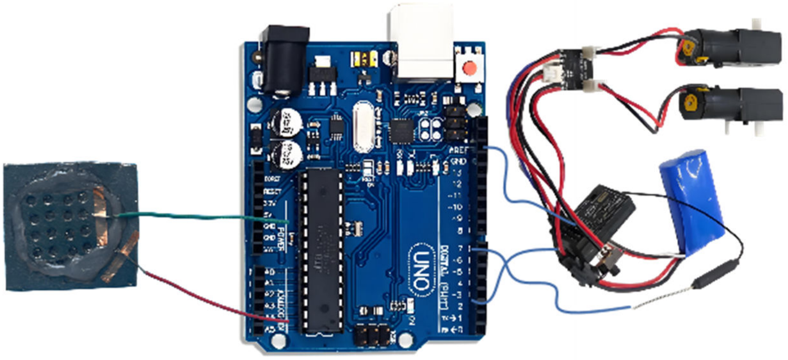

Fig. S27 Image of smart car control system based on MEG device.

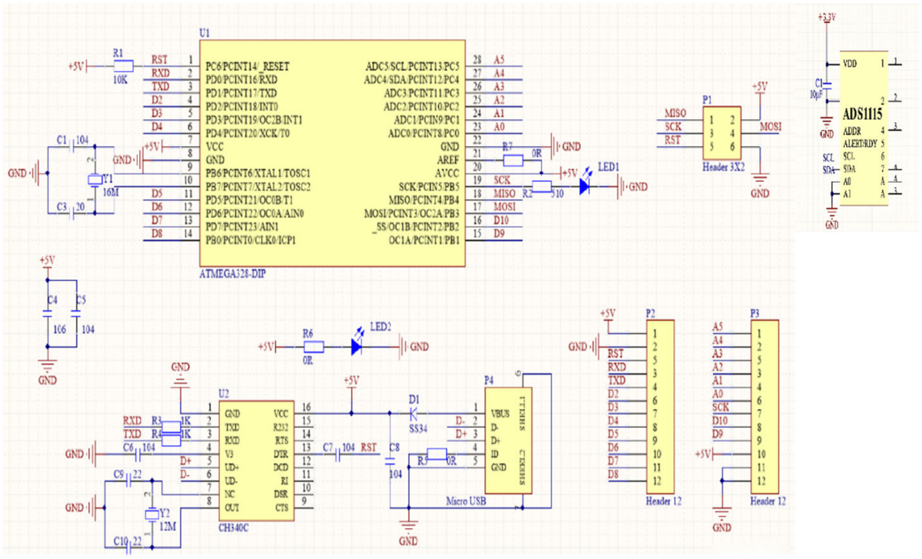

Fig. S28 The detailed circuit schematic of smart car operation system.

Supplementary Table

Table S1 Parameter table for the Flow Simulation.

Material	Property	Value
C electrode	Density(kg/m^3)	2267
	Thermal conductivity(W/m·k)	120
	Specific heat(J/kg·k)	710
	Young’s modulus (Pa)	10000000000
Hydrogel	Density(kg/m^3)	1050
	Thermal conductivity(W/m·k)	0.5
	Specific heat(J/kg·k)	4200
	Young’s modulus(Pa)	10000
	Passion’s ratio	0.45
	Electrical conductivity(S/m)	0.01
Air	Density(kg/m^3)	1.225
	Thermal conductivity(W/m·k)	0.0262
	Specific heat(J/kg·k)	1005
	Relative permittivity	1

Supplemental Video 1: Video showing the command sequences corresponding to “E” and “G” executed and achieved high accuracy in navigation.

Supplemental Video 2: Video showing the circuit connection of the smart car controlled by the MEG devices through the Ardunio port.

Supplemental Video 3: Video showing the car successfully executes two turns and reaches the destination using a sequence of noncontact handwritten commands.
